# Gene Expression of Metalloproteinases and Endogenous Inhibitors in the Lamellae of Dairy Heifers With Oligofructose-Induced Laminitis

**DOI:** 10.3389/fvets.2020.597827

**Published:** 2020-12-23

**Authors:** Jiafeng Ding, Mingxian Shi, Long Wang, Dongdong Qi, Ze Tao, Muhammad A. Hayat, Tao Liu, Jian-tao Zhang, Hongbin Wang

**Affiliations:** ^1^Department of Veterinary Surgery, Northeast Agricultural University, Harbin, China; ^2^Heilongjiang Key Laboratory for Laboratory Animals and Comparative Medicine, Harbin, China

**Keywords:** bovine laminibovine laminitis, pathogenesis, metalloproteinase, TIMP, real-time quantitative PCR, oligofructose overload model

## Abstract

Bovine laminitis leads to huge economic losses and animal welfare problems in the dairy industry worldwide. Numerous studies suggested that several metalloproteinases (MPs) may play vital roles in the failure of epidermal attachment. To the best of our knowledge, the present study is the first to investigate and characterize the gene-level changes in distinct MPs and endogenous inhibitors using oligofructose (OF)-induced bovine laminitis model. The objective of this study was to determine aberrant MPs and related inhibitors of bovine laminitis in gene level, and to provide reasonable directions for the further protein-level research. Twelve normal Chinese Holstein dairy heifers were randomly divided into treatment group (*n* = 6) and control group (*n* = 6). The heifers in the treatment group were administered with OF solutions at a dose of 17 g/kg of body weight via a stomach tube. The heifers were then humanely euthanized when they met the criteria of bovine laminitis. The heifers in the control group were administered with deionized water at a dose of 2 L/100 kg of body weight. They humanely euthanized at 72 h. The gene expressions of MPs and endogenous inhibitors, namely, matrix metalloproteinases (MMPs), A disintegrin and metalloproteinases (ADAMs), and A disintegrin and metalloproteinase with thrombospondin motifs (ADAMTs), and tissue inhibitors of metalloproteinases (TIMPs) in the lamellae from two groups were determined via real-time quantitative PCR. The gene expressions of MMP-2, MMP-9, ADAMTS-4, and ADAMTS-5 significantly increased (*P* < 0.05), whereas that of TIMP-2 significantly decreased (*P* < 0.05) in the treatment group relative to the control group. No significant difference was found in the gene expressions of ADAM-10, ADAM-17, TIMP-1, and TIMP-3. These results indicated that the gene-level imbalanced condition of MPs and their TIMPs may be the basic cause for the failure of epidermal attachment. At the same time, more detailed protein-level studies would be needed to further clarify the roles of MPs and TIMPs in the pathogenesis of bovine laminitis, especially to MMP-2, MMP-9, ADAMTS-4, ADAMTS-5, TIMP-2 as well as related substrates (e.g., aggrecan and versican).

## Introduction

Bovine laminitis results in huge economic losses and animal welfare problems in the dairy industry worldwide (1, 2). This disease may lead to several laminitis-related claw horn disruptions (CHDs), such as sole ulcer, sole hemorrhage, and white line disease (3). Moreover, it can compromise the structural integrity of lamellae, which are composed of two interdigitating layers, dermal lamellae and epidermal lamellae (4). The lamellae connect the third phalanx (P3) to the inner hoof wall within the capsule. The partial or complete separation of dermal and epidermal lamellae leads to rotation and sinking of the P3 within the capsule, as well as severe pain and lameness (5). The interface of the two layers is basement membrane (BM), which is a vital part of extracellular matrix (ECM). The BM destruction and separation was characteristic histological lesions that mainly cause the failure of epidermal attachment in bovine laminitis (6, 7). However, the related molecular mechanism remains unclear.

In clinical practice, bovine laminitis is typically secondary to several inflammatory diseases, including metritis, Gram-negative pleuropneumonia, and ruminal acidosis (8). Experimental models of bovine laminitis have been built by mimicking these clinical conditions. The oligofructose (OF) overload model is more reliable and more intensively utilized than the other induction models (9, 10). The OF-induced models could exhibit the typical characteristic pathophysiological and histological changes as in clinical cases of bovine laminitis (11, 12). Until now, the OF-induced laminitis model has been fully utilized in multiple studies of equine laminitis, but less in the bovine laminitis (13, 14).

Previous studies reported that several MPs play a critical role in the pathogenesis of equine laminitis, especially in BM destruction and ECM remodeling (15–17). Researchers speculated that MPs may also contribute to bovine laminitis (18, 19). However, the MP events has not been investigated in bovine laminitis thus far. In the present study, we firstly determined three classes of MPs and their endogenous inhibitors. The first class of MPs is the classical matrix metalloproteinase (MMP) family, which can degrade various ECM components, especially distinct types of collagens, gelatin, and lamin (20). The second class of MPs is the A disintegrin and metalloproteinase (ADAM) family, especially ADAM-10 and ADAM-17, which are known as “molecular scissors” because they participate in the proteolytic cleavage, or shedding, of the extracellular regions of other transmembrane proteins; hence, they have important roles in the immune system and inflammatory response (21). The last class of MPs is the A disintegrin and metalloproteinase with thrombospondin motifs (ADAMTS) family, which can degrade the structural proteoglycans of cartilage, thus resulting in osteoarthritis (22). Under physiological conditions, these MPs are tightly regulated by specific tissue inhibitors of metalloproteinase (TIMP) to maintain the homeostasis of proteolytic activity of the target tissue (23).

We used an established the OF-induced bovine laminitis model to determine the gene expressions of representative MPs, namely, MMPs, ADAMs, and ADAMTSs, and their specific TIMPs in the lamellae of laminitic heifers. To the best of our knowledge, the present study is the first to investigate and characterize gene-level changes in distinct MPs and endogenous inhibitors using oligofructose (OF)-induced bovine laminitis model. The objective of this study was to determine aberrant MPs and related inhibitors of bovine laminitis in gene level, and to provide reasonable directions for the further protein-level research. Besides, these findings could provide theoretical basis for the future selection of valuable therapeutic targets for this disease.

## Materials and Methods

### Ethics Statement

The protocols of this study were approved by the Animal Ethics Committee of the Northeast Agricultural University (Harbin, Heilongjiang, China). All experimental animals were continuously monitored by investigators to meet the requirements of Animal Welfare Act (2001) (Permission number: SRM-13).

### Experimental Animals

A total of 12 Chinese Holstein dairy heifers were utilized in this study. Their average age, average body weight, and average body condition score (24) was 20.67 ± 3.01 months, 379.71 ± 19.77 kg, and 3.00 ± 0.23, respectively. Before buying them from the Wandashan Dairy Farm (Harbin, Heilongjiang, China), each heifer was carefully examined by routine blood and biochemical tests to ensure that they were clinically healthy and exhibited normal locomotion and posture without any CHDs.

Before acclimation, the claws of all heifers were uniformly trimmed. During the 30-day acclimation period, the heifers were housed in a large animal experimental barn with sufficient water and grass hay supply (7.5% total water-soluble sugar content). The heifers were then trained to accept clinical examinations. After acclimation, the heifers could be led to walk and trot normally by hand and agreed to be examined without any discomfort.

### Model Induction

All heifers were randomly divided into a treatment group (*n* = 6) and a control group (*n* = 6). Before induction, a 0.85 g/kg dose of OF (98% purity; Bailong Biotech, Inc., Dezhou, Shandong, China) was dissolved into warm deionized water (0.1 L/100 kg), then administered to the heifers in the treatment group via a stomach tube twice daily for 3 days (10). Afterwards, similar methods were utilized, a 17 g/kg dose of OF was dissolved into warm deionized water (2 L/100 kg), and administered to these heifers via a stomach tube. The heifers in the control group were administered with 2 L/100 kg warm deionized water only by the same method.

Locomotion assessment and hoof testing were performed at −72, 0 (administration time), 6, 12, 18, 24, 36, 48, 60, and 72 h. Before the locomotion assessment, five licensed veterinarians were carefully trained by watching videos of bovine lameness. They were also required to take lameness-scoring examinations to meet the requirements of this study. During the assessment, all heifers were led by the same investigator to walk and trot along a straight line or a small circle on the same ground. Meanwhile, they were blindly scored by the trained veterinarians according to a 5-point scoring system (Table 1) (25). When a heifer got a score of ≥2 from all veterinarians, it was considered lame. During the hoof testing, a hoof tester was applied on the junction of the axial sole-bulb and at central site of the dorsoabaxial claw wall in four front claws. A suitable pressure was then applied onto the claws to assess the heifers' attempts to withdraw their legs. Their reactions were subjectively classified as none, slight, or marked.

**Table 1:** Locomotion scoring system of dairy cows based on Sprecher et al. (25).

**Lameness score**	**Description**	**Assessment criteria**
1	Normal	Heifer stands and walks with a level-back posture. Its gait is normal
2	Mildly lame	Heifer stands with level-back posture but walks with an arched-back posture. Its gait is normal
3	Moderately lame	Heifer stands and walks with an arched-back posture. Its gait develops a short-striding step with one or more limbs
4	Lame	Heifer stands and walks with an evident arched-back posture. Its gait develops a deliberate step at a time. Heifer favors one or more limbs/feet
5	Severely lame	Heifer demonstrates an inability or extreme reluctance to bear weight on one or more of its limbs/feet

Following the recommendations of previous studies, supportive therapy was provided to the heifers in the treatment group to ensure their welfare and allow comparison of the results with those of previous studies (10). Ringer's acetate (dose of 15 mL/kg of body weight; Heping Animal Medicine, Inc., Harbin, China) and sodium bicarbonate (84 g/L, at a dose of 1.5 mL/kg of body weight; Heping Animal Medicine, Inc., Harbin, China) were quickly administered via jugular infusion at 18 and 24 h. Calcium borogluconate (14 mg Ca/mL, at a dose of 1.4 mL/kg of body weight; Heping Animal Medicine, Inc., Harbin, China) was slowly administered via jugular infusion at 18 h.

### Sample Acquisition

On the basis of the accepted criteria for diagnosing bovine laminitis, a heifer in the treatment group that scored ≥2 in the locomotion assessment and showed consecutive painful reactions in the same claw was humanely euthanized (9, 10, 26). The heifers in the control group were humanely euthanized at 72 h. The lame front limb was rapidly disarticulated from the metacarpophalangeal joint. Several tissue blocks, which consisted of the P3, lamellae tissues, and the central regions of the dorsoabaxial claw wall, were harvested using a band saw. Afterward, the lamellae (~1 cm^2^) were rapidly dissected from the tissue blocks using a lancet (11). Finally, partial samples were immediately frozen in liquid nitrogen and later stored at −80°C.

### Physiological Parameter Measurement

The basic physiological parameters, including heart rate, rectal temperature, respiratory rate, and the pH value of rumen liquid, were measured in the heifers from both groups at −72, 0, 6, 12, 18, 24, 36, 48, 60, and 72 h. Heart rate was recorded using a STAR 8000E patient monitor (Coman Medical Device, Shenzhen, Guangdong, China) three times, and then its mean value was calculated. Rectal temperature was taken using an MC-246 electronic thermometer (Omron, Dalian, Liaoning, China) inserted into the anus. Respiratory rate was determined by observing chest and abdominal contraction and relaxation by three investigators, and then its mean value was computed. The pH value of rumen liquid was measured using an HI 9125 pH meter (Hanna Instruments, Vinsokitt, Rhode Island, USA).

### RNA Isolation and cDNA Synthesis

Total RNA was extracted from the lamellae of the 12 heifers by using the Absolutely RNA Miniprep kit (Stratagende, Inc., LaJolla, CA, USA) according to the manufacturer's instructions. The extracted RNA concentration was measured using a Nanodrop 1000UV-Vis spectrophotometer (Thermo Scientific, Waltham, MA, USA). The absorption rations of all samples met 1.95–2.05 (OD 260/OD 280) and over 2.00 (OD 260/OD 230), indicating high RNA purity. The integrity of RNA samples was verified by 1% agarose gel electrophoresis (Bio-rad Laboratories, Hercules, CA, USA). The concentration of RNA samples was adjusted to 1 μg/μL by measuring optical density. Total RNA (1 μg) was reverse-transcribed using the PrimeScriptTM RT reagent kit with gDNA eraser (Takara, Dalian, Liaoning, China) following the manufacturer's protocols. Complementary DNA (cDNA) was then diluted with DNase/RNase free water (dilution rate: 1:4) (Takara, Dalian, Liaoning, China) and stored at −20°C.

### Real-Time Quantitative PCR (qPCR)

The primer sets utilized in this study were designed to recognize and amplify conserved nucleotide sequences encoding related bovine MPs and TIMPs. The primers used for housekeeping genes (β-actin, GAPDH, and UXT) were referred from previous studies (27, 28). Primers for MMP-2, MMP-9, ADAM-10, ADAM-17, ADAMTS-1, ADAMTS-4, ADAMTS-5, TIMP-1, TIMP-2, and TIMP-3 were designed using the Primers 5 software (White Institute, Cambridge, MA, USA). The specificity of selected primer sequences was verified using the BLAST computer program from the National Center for Biotechnology Information database (Bethesda, Maryland, USA).

Real-time quantitative PCR (RT-qPCR) was performed using the LightClycler 480 RT-PCR system (Roche, Indianapolis, Indiana, USA) with fluorescence detection of SYBR Premix Ex TaqTM II (Takara, Dalian, Liaoning, China). The PCR mixture (20 μL) consisted of 2 μL sample cDNA and 18 μL PCR master mix. Thereafter, the PCR master mix contained 1.6 μL of mixed primer solutions (10 μM each of forward and reverse primers), 6.4 μL DNase/RNase free water, and 10 μL SYBR green dye. Amplification conditions were set by the classic four-step method: denaturation of temple DNA at 95°C for 1 min; 40 cycles of amplification (quantification analysis model) at 95°C for 5 s and 60°C for 1 min; one cycle of melting (melting curve analysis model) at 95°C for 5 s, 60°C for 1 min, and then at 95°C; and one cycle of cooling at 50°C for 30 s. All measurements were performed in triplicate.

The values of cycle threshold (Ct) crossing were calculated using the Light Cycler 480 software (version 1.5.0; Roche, Indianapolis, IN, USA). All amplified cDNA products were verified by 1% gel electrophoresis and melting curve analysis. Template DNA (10-fold serial dilution) was used to generate standard curves and calculate the efficiency for each PCR reaction (Table 2). A reverse-transcription negative blank of each sample and a no-template blank were utilized as negative controls. The Ct value of each targeted mRNA was normalized to the geometric mean of three housekeeping genes, and the data were calculated according to the 2^−ΔΔCt^ method and considering quantitative PCR efficiency (19, 28, 29).

**Table 2:** Gene, sequence, amplification size, and efficiency of the primers used for RT-qPCR.

**Gene**	**Polarity**	**Sequence (5^**′**^ → 3^**′**^)**	**Size (bp)**	**Efficiency (%)**	**GenBank ID**
**Matrix metalloprotease (MMP)**					
MMP-2	Forward	GCTGTGTACGAAGACCCACA	289	96	NM_174745.2
	Reverse	CCAGGTTATCAGGGATGGCG			
MMP-9	Forward	GGGTAAGGTGCTGCTGTTCA	136	105	NM_174744.2
	Reverse	CTGAAAGATGTCGTGCGTGC			
**A disintegrin and metalloprotease (ADAM)**					
ADAM-10	Forward	GCAGTCCAAGTCAAGGTCCC	241	92	NM_174496.3
	Reverse	GCACAGGTACACTCCTCCAA			
ADAM-17	Forward	AGTCACGGAGGTGTTTGTCC	193	91	XM_010810303.3
	Reverse	CACATTCCGCCAGACCATCT			
**A disintegrin and metalloprotease with thrombospondin motifs (ADAMTS)**					
ADAMTS-1	Forward	CAGACCGACAAGGAGCACTT	147	97	NM_001101080.1
	Reverse	TTTTCCTCCGTTCTTCGGCA			
ADAMTS-4	Forward	TGCCAGACTAAGCACTCACC	116	103	NM_181667.1
	Reverse	GCCTGTGGGACATTGAAAGC			
ADAMTS-5	Forward	CACAAAGGTGGTCGGAACCT	120	95	NM_001166515.1
	Reverse	AATCTGGTCTGGTCCTTGGC			
**Tissue inhibitors of metalloprotease (TIMP)**					
TIMP-1	Forward	GGCCTTCTGCAACTCCGAT	133	106	XM_005228099.2
	Reverse	ATCCCTCAAGGCACTGAACC			
TIMP-2	Forward	GATCCGAGCCAAGGTGGTAG	111	97	NM_174473.4
	Reverse	TGGGGCATCTTGGTGAATCC			
TIMP-3	Forward	TGATCCGAGCCAAGGTGGTA	111	93	NM_174473.5
	Reverse	GGGGCATCTTGGTGAATCCT			
**Housekeeping gene**					
ACTB	Forward	ACTTGCGCAGAAAACGAGAT	123	97	NM_173979.3
	Reverse	CACCTTCACCGTTCCAGTTT			
GAPDH	Forward	GGGTCATCATCTCTGCACCT	176	95	NM_001034034.2
	Reverse	GGTCATAAGTCCCTCCACGA			
UXT	Forward	TGTGGCCCTTGGATATGGTT	101	101	NM_001037471.2
	Reverse	GGTTGTCGCTGAGCTCTGTG			

### Data Analysis

Data analysis was performed using GraphPad Prism software (version 7.04; GraphPad Software, Inc., San Diego, CA, USA). According to the results of D'Agostino and Pearson and Shapiro–Wilk normality tests, the data of physiological parameters (i.e., heart rate, rectal temperature, respiratory rate, and the pH value of rumen liquid) fulfilled the assumption of a Gaussian distribution. The data were analyzed by two-way ANOVA (Bonferroni's multiple comparisons test for the treatment and control groups within a time point; Tukey's multiple comparisons test for the treatment group relative to the baseline value [time 0]). Similarly, the data of relative gene expression fulfilled the assumption of a Gaussian distribution according to the results of Shapiro–Wilk normality test. The data were analyzed by Student's *t*-test. In this study, *P* < 0.05 was considered significant. All data are presented as mean ± standard deviation (SD).

## Results

### Clinical Manifestations and Physiological Data

**Figure 1:**
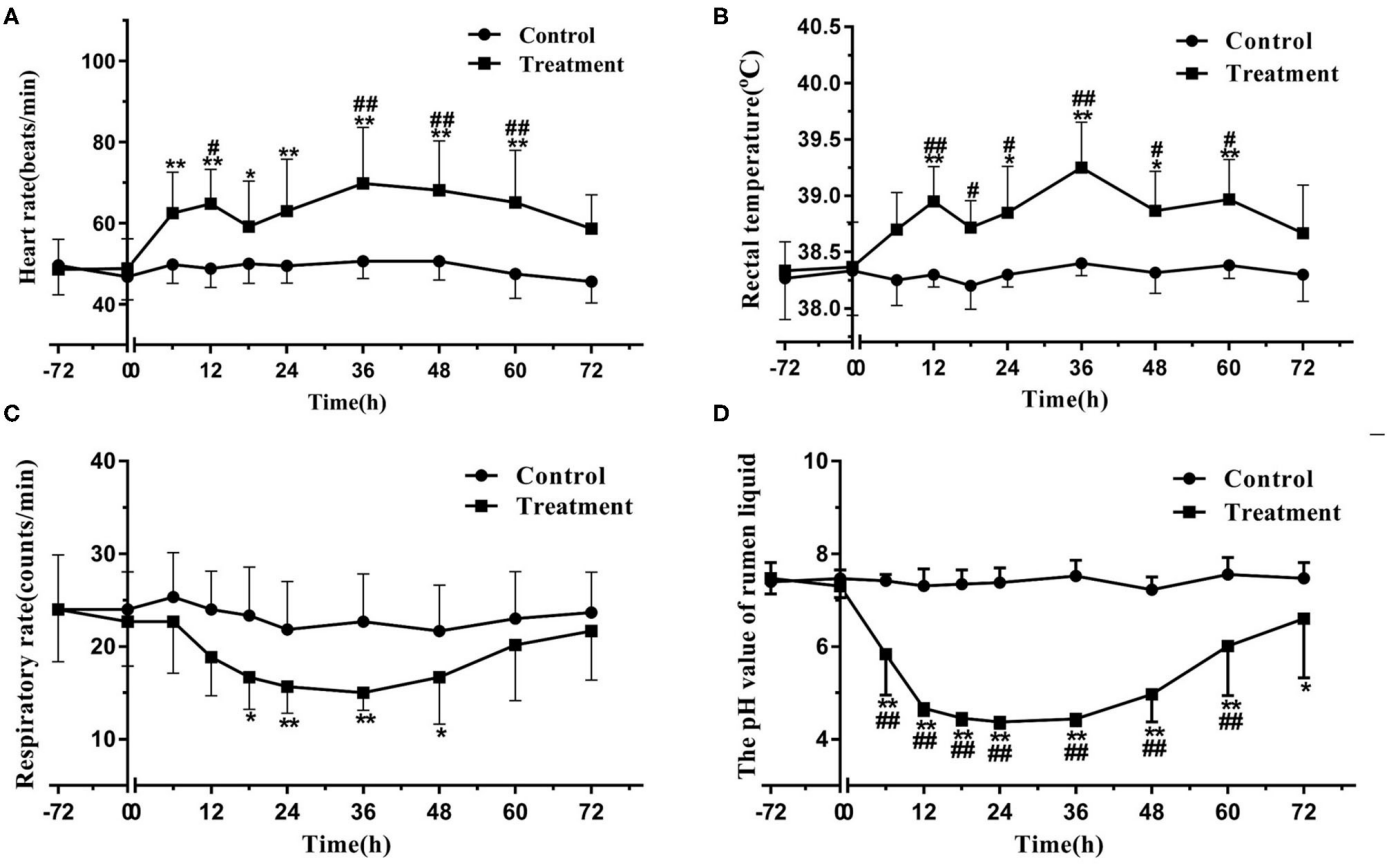
Changes in heart rate **(A)**, rectal temperature **(B)**, respiratory rate **(C)**, and pH value of rumen liquid **(D)** in the heifers with oligofructose-induced laminitis. Within a time point, the values of the treatment group significantly differed from those of the control group, indicated as ^#^*P* < 0.05 and ^*##*^*P* < 0.01; within the treatment group, the values of distinct time points significantly differed from the baseline value (time 0), indicated as * *P* < 0.05 and ** *P* < 0.01.

The heifers in the treatment group developed evident symptoms of acute rumen acidosis and systemic inflammatory response syndrome, including depression, anorexia, watery diarrhea, tachycardia, bradypnea, pyrexia, and decreased pH value of rumen liquid. By contrast, the clinical manifestations of the heifers in the control group were normal. The heart rate of the heifers in the treatment group significantly increased (*P* < 0.05) relative to the baseline (time 0) at 6–60 h and to that of the heifers in the control group at 12 and 36–60 h. The rectal temperature of the heifers in the treatment group significantly increased (*P* < 0.05) relative to the baseline at 12 and 24–60 h and to that of the heifers in the control group at 12–60 h. The respiratory rate of the heifers in the treatment group significantly decreased (*P* < 0.05) relative to the baseline at 18–48 h. The pH value of rumen liquid of the heifers in the treatment group significantly decreased (*P* < 0.05) relative to the baseline at 6–72 h and to that of the heifers in the control group at 6–60 h. The data on physiological parameters are illustrated in Figure 1.

In the treatment group, the heifers' locomotion scores were 1 (*n*ormal) at 0 h, and they initially developed signs of lameness at 36 h. At 72 h, the locomotion scores of all heifers were ≥2, and each of them had a consecutive painful reaction in the same claw. By contrast, in the control group, the heifers' locomotion scores were 1 at selected time points and did not exhibit consecutive painful reactions in hoof testing examination.

### Gene Expressions of Laminar MMPs in Bovine Laminitis

**Figure 2:**
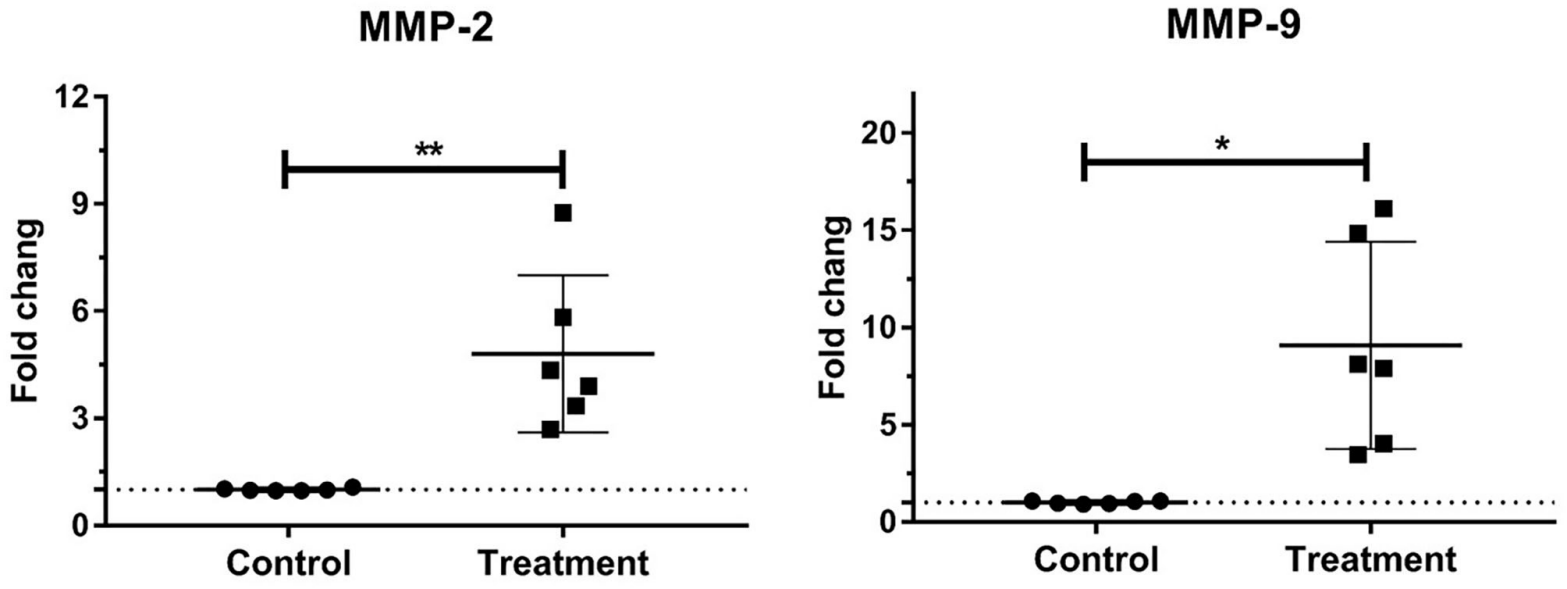
Mean fold changes in MMP-2 and MMP-9 mRNA expression following oligofructose-overload administration in dairy heifers. Significant increases in the treatment group relative to those in the control group, indicated as *P < 0.05 and **P < 0.01.

The gene expressions of laminar MMP-2 (*P* < 0.01) and MMP-9 (*P* < 0.05) significantly increased in the treatment group relative to those in the control group (Table 3, Figure 2).

**Table 3:** Fold changes in the gene expressions of laminar metalloproteases and their inhibitors in the heifers with oligofructose-induced laminitis.

**Metalloprotease**	**Fold change (mean ± SD)**	***P-*value**
**Matrix metalloprotease (MMP)**		
MMP-2	4.81 ± 2.20 ^↑^	0.0082
MMP-9	3.46 ± 3.89 ^↑^	0.0139
**A disintegrin and metalloprotease (ADAM)**		
ADAM-10	0.86 ± 0.37 ^NS^	0.3901
ADAM-17	1.03 ± 0.58 ^NS^	0.9147
**A disintegrin and metalloprotease with thrombospondin motifs (ADAMTS)**		
ADAMTS-1	1.18 ± 0.57 ^NS^	0.4983
ADAMTS-4	9.20 ± 2.18 ^↑^	0.0002
ADAMTS-5	4.90 ± 1.31 ^↑^	0.0022
**Tissue inhibitors of metalloprotease (TIMP)**		
TIMP-1	0.81 ± 0.30 ^NS^	0.1866
TIMP-2	0.43 ± 0.10 ^↓^	<0.0001
TIMP-3	0.73 ± 0.37 ^NS^	0.1303

*Values displayed are from fold changes in the treatment group relative to those in the control group; NS, non-significant change in values; ↑, significant increase in values; ↓, significant decrease in values*.

### Gene Expressions of Laminar ADAMs in Bovine Laminitis

**Figure 3:**
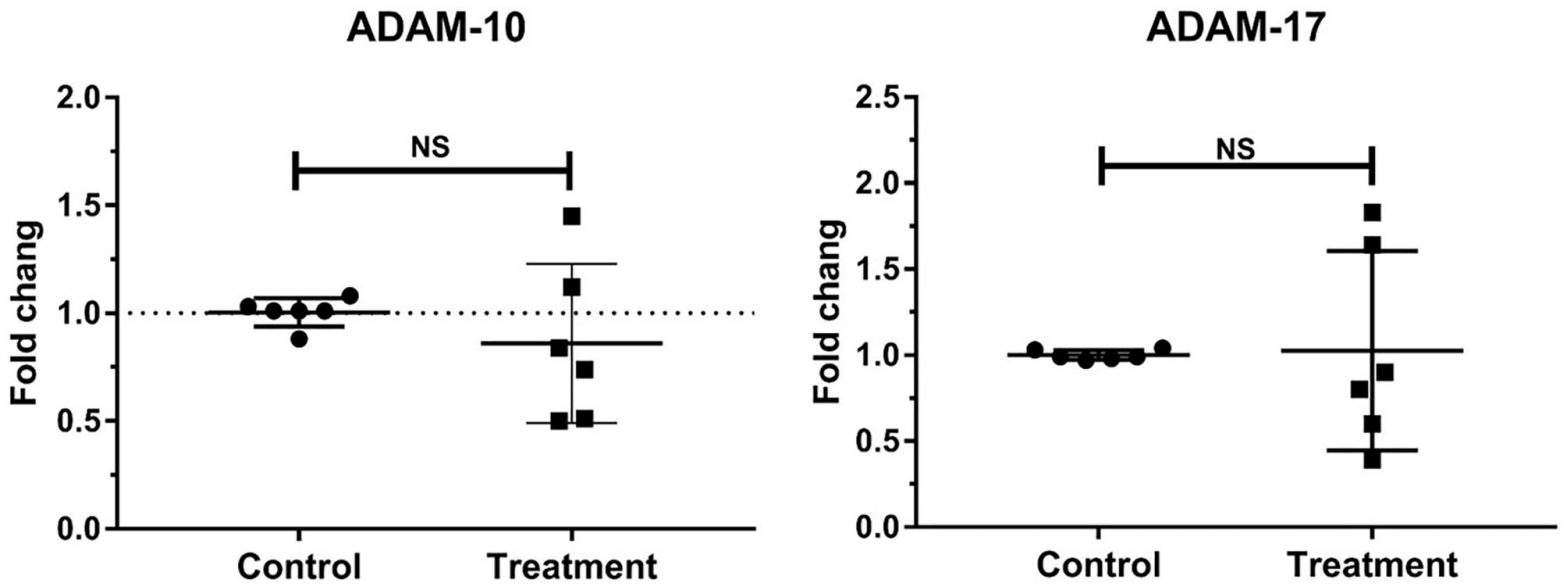
Mean fold changes in ADAM-10 and ADAM-17 mRNA expression following oligofructose-overload administration in dairy heifers. No significant differences in the treatment group relative to those in the control group are indicated as NS.

No change in the gene expressions of laminar ADAM-10 and ADAM-17 was observed in the treatment group relative to that in the control group (Table 3, Figure 3).

### Gene Expressions of Laminar ADAMTs in Bovine Laminitis

**Figure 4:**
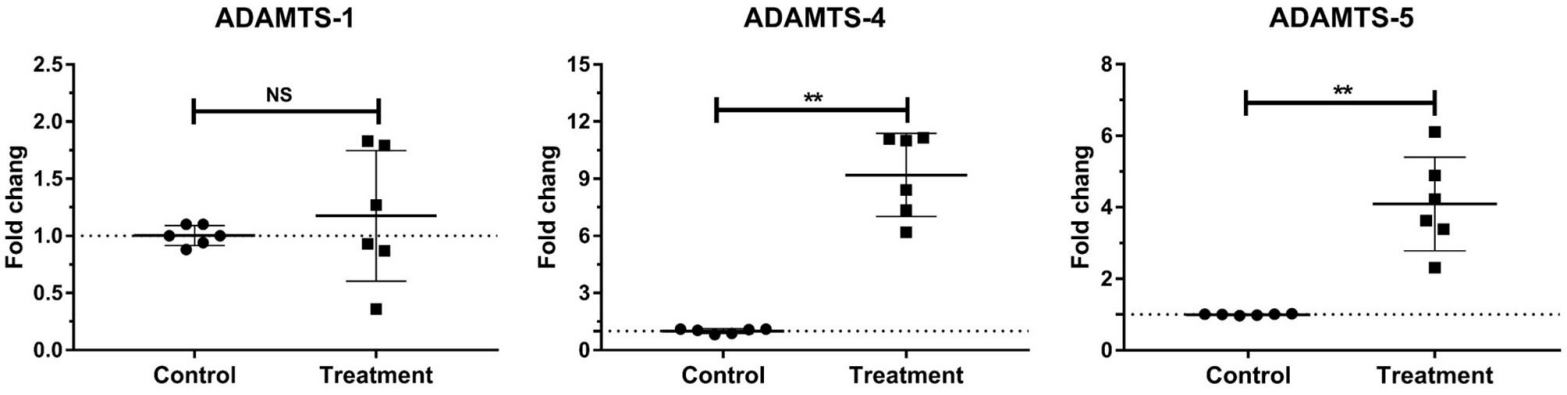
Mean fold changes in ADAMTS-1, ADAMTS-4, and ADAMTS-5 mRNA expression following oligofructose-overload administration in dairy heifers. The values of the treatment group significantly differed from those of the control group values, indicated as **P < 0.01; no significant differences indicated as NS.

The gene expressions of laminar ADAMTS-4 (*P* < 0.001) and ADAMTS-5 (*P* < 0.01) significantly increased in the treatment group relative to those in the control group. No change was observed in the gene expression of laminar ADAMTS-1 (Table 3, Figure 4).

### Gene Expressions of Laminar TIMPs in Bovine Laminitis

**Figure 5:**
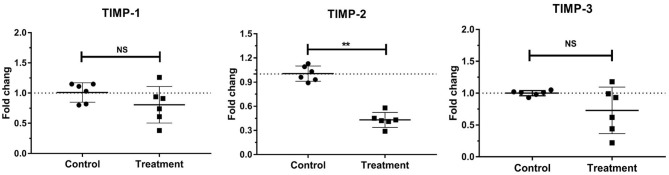
Mean fold changes in TIMP-1, TIMP-2, and TIMP-3 mRNA expression following oligofructose-overload administration in dairy heifers. The values of the treatment group significantly differed from those of the control group values, indicated as **P < 0.01; no significant differences indicated as NS.

The gene expressions of laminar TIMP-2 significantly decreased (*P* < 0.001) in the treatment group relative to those in the control group. No change was observed in the gene expressions of laminar TIMP-1 and TIMP-3 (Table 3, Figure 5).

## Discussion

Considering the important role of MPs and TIMPs in BM destruction and separation of equine laminitis, numerous researchers inferred that disordered MP events may also contribute to bovine laminitis (18, 19). However, no study has reported this event thus far. Thus, we established a previously described (and reliable) bovine laminitis model, and then determined the gene expression of representative MPs and their endogenous inhibitors. To our knowledge, the present study is the first to investigate and characterize gene-level changes of MP events in OF-induced bovine laminitis.

Bovine laminitis was initially described as a diffuse aseptic inflammation of the dermal lamellae of the claw (30). Afterward, researchers proposed a broader definition of bovine laminitis as a systemic disease with local manifestations in the claw (8). In clinical practice, bovine laminitis is typically secondary to severe systemic inflammatory diseases, especially rumen acidosis, septic pleuropneumonia, metritis, etc. We adopted the OF-induced laminitis model in this study because it mimics well the practical conditions in modern dairy farms, where high-energy feeding often leads to the occurrence of rumen acidosis in dairy cows (31). Moreover, this model could exhibit the typical characteristic pathophysiological and histological changes as in clinical cases of bovine laminitis. Besides, this model was easier to operate and successfully establish experimental bovine laminitis, and had better repeatability than other induction models (10).

An overloaded ingestion of OF (a type of non-structural carbohydrate), which can rapidly reduce the pH of rumen liquid, compromises intestinal barrier functions and results in the death of numerous Gram-negative bacteria. The free lipopolysaccharide (LPS) translocates from the digestive tract into the peripheral circulation (32), leading to systemic inflammatory responses and multiple organ damage, including laminitis (33), rumenitis (34), and synovitis (35).

Lamellae tissues are critical for maintaining proper P3 orientation and transferring mostly mechanical forces to the hoof because they link the P3 to the hoof capsule (36). Lamellae consist of interconnected epidermal lamellae and dermal lamellae, as well as a contact interface that is BM. BM destruction and detachment are the characteristic histological lesions that contribute to the failure of epidermal attachment in bovine laminitis (11). Numerous complex components are present around BM, including collagen, laminin, MPs, versican, aggrecan, and keratin (37). MPs are the primary proteases involved in ECM remodeling under physiological and pathological conditions (38, 39). Thus, we can reasonably speculate that disordered proteolytic events involving MPs and other components may occur in the progression of bovine laminitis.

In our published literature, we have already proved that the bovine laminitis was successfully established using the OF-induced model, and laminitic heifers developed characteristic histological lesions in the lamellae, including BM destruction and detachment, stretching of epidermal lamellae and changes in basal cell morphology (33). Herein, we utilized the same batch of lamellae tissue as that literature, then conducted further gene-level measurement of representative MPs and TIMPs.

In the present study, the gene expression of laminar MMP-2 and MMP-9 significantly increased in the OF-induced heifers. These results were consistent with those of multiple studies on equine laminitis (15, 16) and those of a study on dairy cows with sole ulcer (a type of laminitis-related lesion) (19), suggesting that these MMPs may also be deeply implicated in bovine laminitis.

One of the primary mechanisms of MMP-2/MMP-9 regulation is at the level of transcription (20, 39), especially they could be induced response to multiple stimuli, including proinflammatory cytokines, transforming growth factors, and LPS (40). In our previous studies, we utilized the same batch of lamellae samples as this study, and reported the increased gene expression of multiple proinflammatory mediators (33). These results may provide a possible explanation to the increases in MMP-2 and MMP-9 expression observed in the present study. Moreover, several signaling pathways, such as nuclear factor kappa B, mitogen-activated protein kinases, and signal transducers and activators of transcription, also participate in the transcriptional regulation of MMPs (41).

Generally, the transcriptional-level MMP-2 and MMP-9 are important basis for further protein-level expression of proenzyme MMP-2 (proMMP-2) and proenzyme MMP-9 (proMMP-9) as well as MMP-2 and MMP-9 (20, 39). Both MMP-2 (gelatinase-A) and MMP-9 (gelatinase-B) play a key role in the remodeling of collagenous ECM. MMP-9 can especially degrade denatured collagen and gelatin, native type I collagen of ECM, and native type IV collagen of BM, as well as laminin components in lamellae (42). By comparison, MMP-2 can degrade distinct native collagens and laminin, as well as aggrecan, fibronectin, and vitronectin in ECM (43).

MMP-2 and MMP-9 can be expressed by various cell types, such as endothelial cells, fibroblasts, keratinocytes, and monocytes in the case of MMP-2, and granulocytes, macrophages, and osteoclasts in the case of MMP-9 (39). In equine laminitis, the concentration of laminar proMMP-9 is strongly correlated with neutrophil migration into the lamellae and myeloperoxidase (MPO) content, suggesting that proMMP-9 may be released from or induced by neutrophils. By contrast, the concentration of proMMP-2 has no correlation with MPO content and proMMP-9, suggesting that proMMP-2 may be expressed by innate cells in the lamellae rather than by migrant inflammatory leukocytes (44). Using the same samples, we found that an increase in MPO content also has a strong positive correlation with an increase in proMMP-9 rather than in proMMP-2 in bovine laminitis. These results indicated that proMMP-9 may be released from migrated neutrophils, whereas proMMP-2 may be expressed by innate cells in the lamellae, such as fibroblasts and keratinocytes.

The conversion from proMMP-9 to MMP-9 primarily involves three pathways: proteolytic removal of proMMP-9 propeptide, binding of proMMP-9 to active substrates, and oxidative modification of proMMP-9 (45). Meanwhile, proMMP-2 is converted to MMP-2 via two pathways: induction by the MT-MMP/TIMP-2 complex (but this way has been suspected in equine laminitis); and processing by neutrophil-derived proteases, especially elastase, cathepsin-G, and proteinase-3 (46). From previous studies of equine laminitis, only inactive proMMP-9 and MMP-2 were increased in the lamellae of OF-induced experimental model as well as naturally clinical cases (44, 47). More protein-level studies (e.g., using native zymography technology) should be performed to confirm the changes of MMP-9/MMP-2 activity in these samples.

Aside from gelatinases, researchers studying laminitis also focus on ADAMs, a family of membrane-anchored enzymes, because they can regulate distinct cellular functions, such as proteolysis, cell adhesion and migration, and cellular signaling (48). ADAM-10 and ADAM-17 are regarded as important research hotspots because of their vital roles in immunity and inflammatory response (49).

In the present study, the gene expressions of laminar ADAM-10 and ADAM-17 were stable in the heifers with OF-induced laminitis relative to those in the control group. Previous studies obtained similar results in OF-induced and naturally acquired equine laminitis (15). Notably, these results were unexpected because ADAM-10 and ADAM-17 reportedly participate in nearly all central events of inflammation response, including myelopoiesis, inflammatory cytokine production, migration, phagocytosis, and antigen-presenting functions (21). Using the same samples, we reported in a previous study that the gene expression of laminar tumor necrosis factor-α is stable in bovine laminitis (33), maybe it is a reasonable explanation for the stable ADAM-17 gene expression observed herein because ADAM-17 can regulate the cleavage of membrane-bound TNF into soluble active TNF (50, 51). Another possible explanation to these results is that the low level of inflammatory response actually occurred in this laminitis model: the OF-induced heifers experienced transient fever and tachycardia and then their lameness symptoms eventually disappeared, indicating that this model induced a relatively mild laminitis (9, 10).

ADAMTS, the secreted-type metalloprotease of the ADAMTS superfamily, has catalytic activity and participates in ECM remodeling because its substrates are the primary structural components of ECM (52, 53). Furthermore, ADAMTS protease has the propensity to adhere to the pericellular matrix of basal cells, thereby affecting cell adhesion and survival (54). Thus, several researchers speculate that ADAMTS may greatly contribute to the pathogenesis of bovine laminitis (16, 55).

ADAMTS-1 was the first cloned protease in the ADAMTS superfamily (56). This protease is involved in regulating organ morphology and functions, vascular biology, and degenerative intervertebral disc diseases similar to degenerative osteoarthritis (OA) (57–59). However, in the present study, the gene expression of laminar ADAMTS-1 was stable, suggesting that it may not participate in the development of this disease.

By contrast, the gene expressions of laminar ADAMTS-4 and ADAMTS-5 significantly increased in the heifers with OF-induced laminitis. Using the same sample, we previously reported increased laminar IL-1 and IL-6 gene expression (33). An increase in the gene expression of these interleukins may induce increases in ADAMTS-4 and ADAMTS-5 expression because IL-1 and IL-6 reportedly contribute to the induction of ADAMTS-4 and ADAMTS-5 in the cartilage (60). Moreover, ADAMTS-4 and ADAMTS-5 purportedly can affect tissue growth and upkeep, vascular biology, and OA progression (61). They are considered as the major aggrecanases in the development of OA because they can degrade the structural aggrecans of cartilage ECM. With regard to bovine laminitis, aside from aggrecans, multiple other glycoproteins and proteoglycans also exist around BM and basal epidermal cells, such as versican, brevican, and biglycan (37, 62, 63). Notably, these components can also be cleaved by ADAMTS-4 and ADAMTS-5 (64).

Similar results were obtained by a previous study on equine laminitis, which indicated that changes in ADAMTS-4 and versican may arise from physiological changes in basal epidermal cells (55). These changes eventually lead to the separation between BM and epidermal lamellae. On the basis of evident changes in morphology and biological condition of basal epidermal cells, the separation between BM and epidermal lamellae, the loss of glycoproteins and proteoglycans around BM (12, 26), and the increase in gene expressions of ADAMTS-4 and ADAMTS-5 and their special biological properties, we hypothesize that ADAMTS-4 and ADAMTS-5 play a key role in bovine laminitis. Additional direct evidence is needed to verify our hypothesis. In follow-up studies, these valuable parameters, including the ADAMTS-4 and ADAMTS-5, their substrates (e.g., aggrecan and versican), and the cleaved fragments of their substrates, should be detected at the protein level.

TIMPs are small endogenous proteins that contain an N-terminal inhibitory domain that binds to the active domains of MMPs, ADAMs, and ADAMTSs. Moreover, TIMPs inhibit the activities of these MPs. Therefore, TIMPs are the vital regulators of ECM turnover, tissue remodeling, and cellular signaling (65). Aside from inhibiting MP activities, TIMPs also perform various biological activities, such as promoting cell proliferation, antiangiogenic activity, and synaptic plasticity activities associated with motor dysfunction (66). The TIMP family has four members, of which three were selected in the present study. TIMP-3 exhibited the broadest inhibition array of MPs because it could inhibit multiple members of the ADAM and ADAMTS families (Table 4).

**Table 4:** Summary of inhibition relationships between the selected TIMPs and MPs in the present study.

**Property**	**TIMP-1**	**TIMP-2**	**TIMP-3**
proMMP interaction	proMMP-9	proMMP-2	proMMP-2, 9
MMP inhibition	MMP-9	MMP-2, 9	MMP-2, 9
Other MP inhibition	ADAM-10		ADAM-10, 17; ADAMTS-1, 4, 5

*TIMP, tissue inhibitors of metalloproteinase; MP, metalloproteinase; MMP, matrix metalloproteinase; ADAM, A disintegrin and metalloproteinase; ADAMTS, A disintegrin and metalloproteinase with thrombospondin motifs*.

The present study observed that the gene expression of laminar TIMP-2 significantly decreased in the heifers with OF-induced laminitis relative to that in the control group. By contrast, the gene expressions of TIMP-1 and TIMP-3 were stable, suggesting that TIMP-2 may specifically participate in the pathogenesis of bovine laminitis. Recently, our research group finished using the reverse zymography assays to determine the activity of TIMP-1 and TIMP-2, finally found the reduced protein-level TIMP-2 in the OF-induced laminitic heifers (Li, unpublished results). This finding may possibly explain the increases in MMP-2 and MMP-9 activities, because reduced TIMP-2 could decrease its inhibition to MMP-2 and MMP-9 activities. As mentioned previously, proMMP-2 is converted to MMP-2 via the induction of the MT-MMP/TIMP-2 complex. However, in consideration of the decrease in TIMP-2 expression and the increase in MMP-2 expression, this pathway may be not the primary route in bovine laminitis. Other studies reported similar findings (16).

A decrease in the gene expression of laminar TIMP-2 was also found in horses with OF-induced laminitis and dairy cows with sole ulcer (15, 19). This result suggested that TIMP-2 may play a similar role in the laminitis of distinct animal species. Previous studies reported that a decrease in TIMP-2 expression initiates the induction of equine laminitis as observed in the initial period of disease progression (16). Accordingly, researchers regard TIMP-2 as a potential therapeutic target for laminitis.

Stable gene expressions of laminar TIMP-1and TIMP-3 were also observed in OF-induced equine laminitis (15). Given the stability of ADAM-10 and ADAM-17 expression, the stable gene expressions of TIMP-1 and TIMP-3 seem reasonable because they are potent inhibitors that regulate the induction and activation of these two ADAMs (21, 67). Although TIMP-3 can finely inhibit the proteolytic activities of ADAMTS-4 and ADAMTS-5 (68, 69), the present study indicated that the stable TIMP-3 expression evidently had a weak effect on the increase in ADAMTS-4 and ADAMTS-5 expression and BM destruction, suggesting that this inhibitor does not play an important role in the development and progression of this disease.

In conclusion, this study reported the gene expressions of three classes of laminar MPs and their inhibitors in the heifers with OF-induced laminitis. We found that the gene expressions of lamellar gelatinase (MMP-2 and MMP-9), ADAMTs (ADAMTS-4 and ADAMTS-5), and an endogenous inhibitor (TIMP-2) was disordered in laminitic heifers. These results indicated that the gene-level imbalanced condition of MPs and their inhibitors may be the basic cause for the BM detachment from epidermal lamellae. This study contributes to our understanding of gene-level MP events in bovine laminitis. At the same time, more detailed protein-level studies would be needed to further clarify the roles of MPs and TIMPs in the pathogenesis of bovine laminitis, especially to MMP-2, MMP-9, ADAMTS-4, ADAMTS-5, TIMP-2 as well as related substrates (e.g., aggrecan and versican).

## Data Availability Statement

The original contributions presented in the study are included in the article/Supplementary Material, further inquiries can be directed to the corresponding author.

## Ethics Statement

The animal study was reviewed and approved by Animal Ethics Committee of the Northeast Agricultural University (Harbin, Heilongjiang, China) (Permission number: SRM-13).

## Author Contributions

JD designed and performed this study, analyzed data, and wrote the manuscript. MS, LW, DQ, ZT, and MH joined the animal experiments and contributed to the seminar discussions. TL, J-tZ, and HW provided valuable guidances. All authors approved the final version of the manuscript.

## Conflict of Interest

The authors declare that the research was conducted in the absence of any commercial or financial relationships that could be construed as a potential conflict of interest.
