# Supplementary material for: Gene Expression of Metalloproteinases and Endogenous Inhibitors in the Lamellae of Dairy Heifers With Oligofructose-Induced Laminitis
Source: Front Vet Sci. 2020 Dec 23;7:597827. doi: 10.3389/fvets.2020.597827 (PMC7786368; doi:10.3389/fvets.2020.597827)
Supplement: Supplementary file 1 [file Data_Sheet_1.docx]

**SUPPLEMENTARY MATERIAL**

**Contents**

1. CERTIFICATE OF ENGLISH EDITING. Language company helped us correct the writing of this manuscript, then we carefully checked and modified it, aiming to let reviewers and other readers easily read it.
2. Video 1. The heifers in the treatment group were induced laminitis after the administration of oligofructose (OF) overload.
3. Video 2. The heifers in the treatment group refused to move after the administration of OF overload, even investigators led them with a big [strength](javascript:;).
4. Picture 1. The hoof band of heifers became red and swell after the administration of OF overload.
5. Picture 2. The process for collecting lamellae samples using a band saw.
6. Picture 3. The process for collecting lamellae samples using a band saw.
7. Picture 4. Basic characteristics of collected rumen liquids from heifers with the OF-induced laminitis.
