# Supplementary figures and images for: Gene Expression of Metalloproteinases and Endogenous Inhibitors in the Lamellae of Dairy Heifers With Oligofructose-Induced Laminitis
Source: Front Vet Sci. 2020 Dec 23;7:597827. doi: 10.3389/fvets.2020.597827 (PMC7786368; doi:10.3389/fvets.2020.597827)

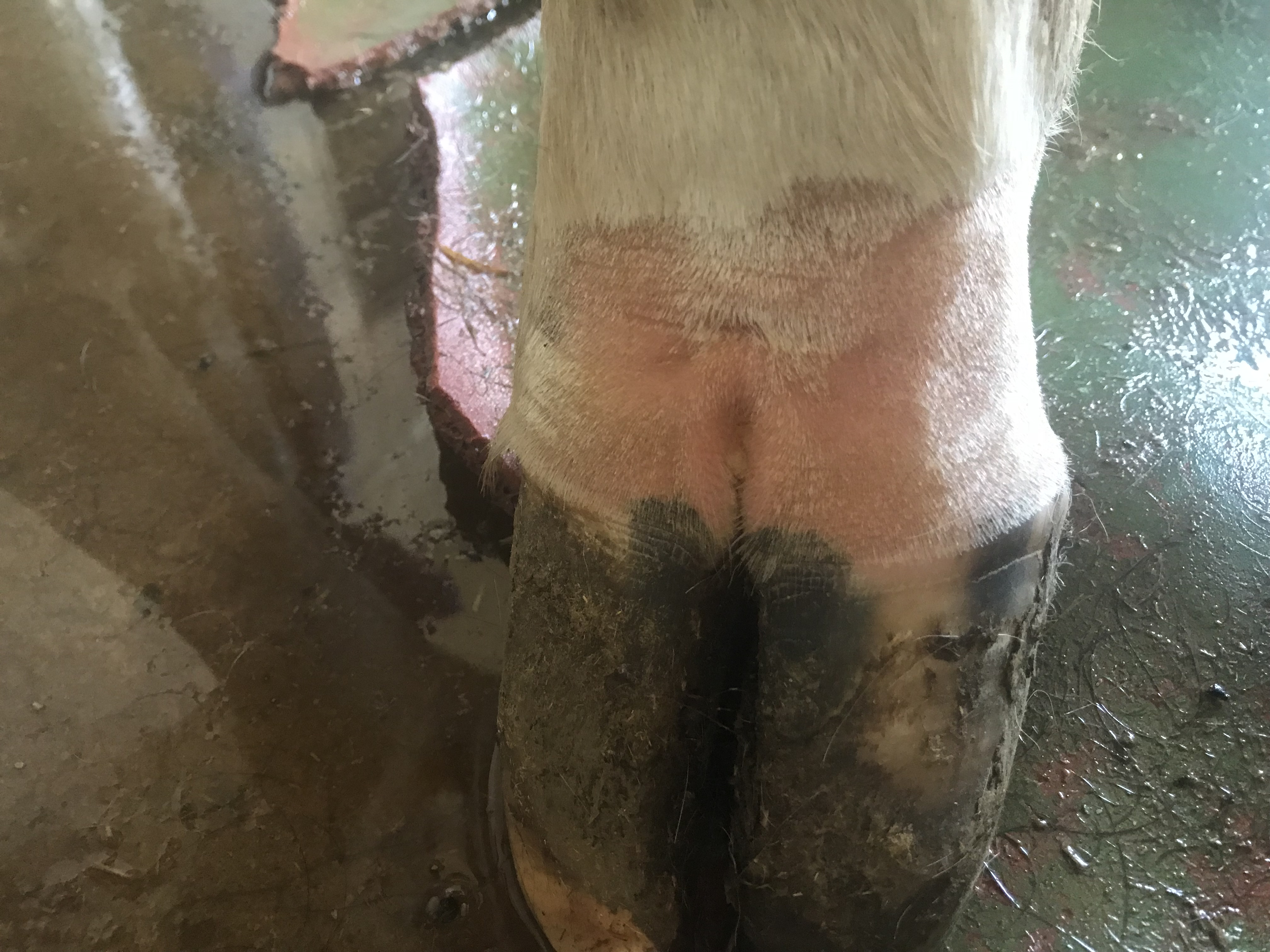

Supplement: Supplementary file 4 [file Image_1.JPEG]

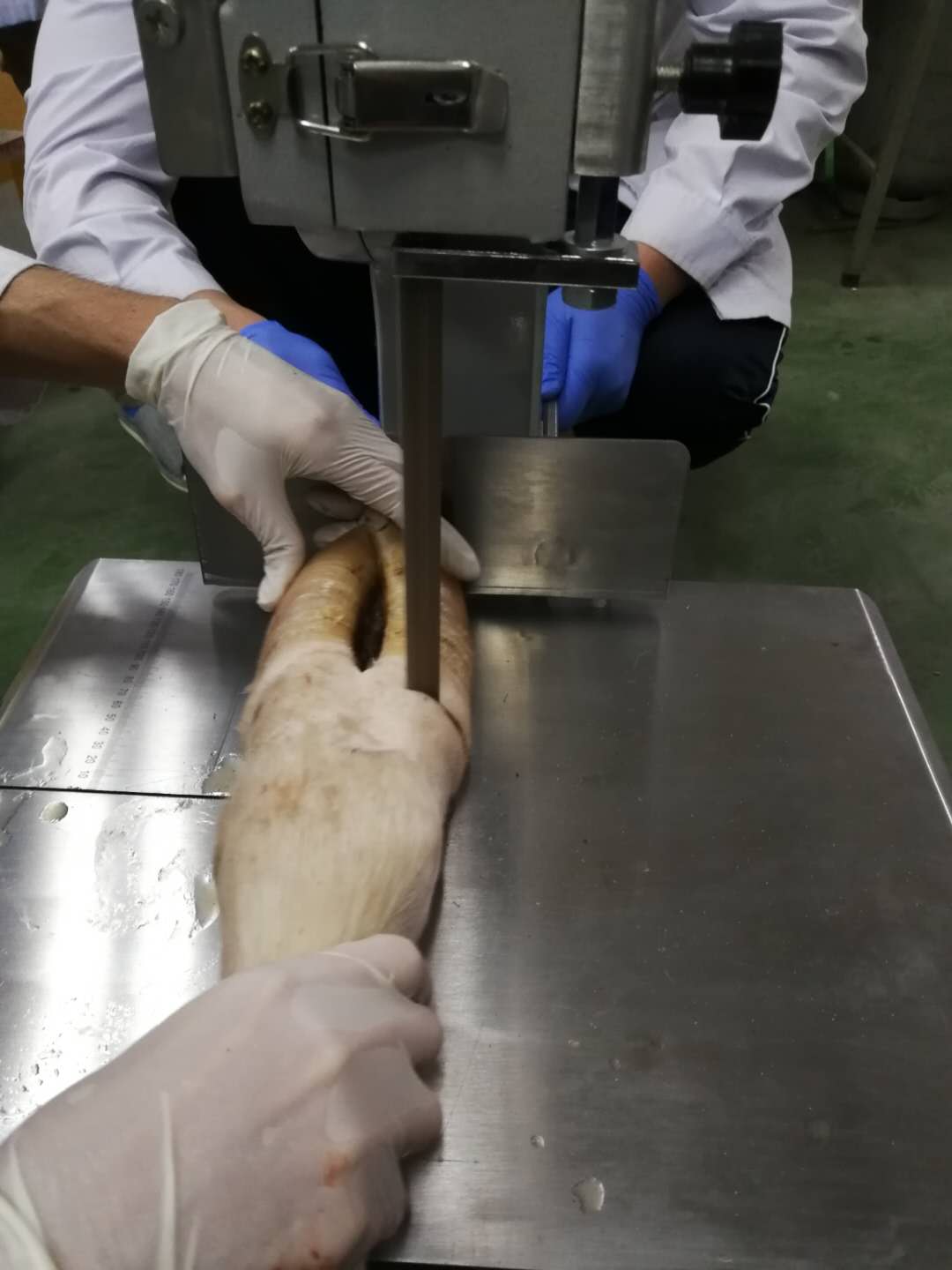

Supplement: Supplementary file 5 [file Image_2.JPEG]

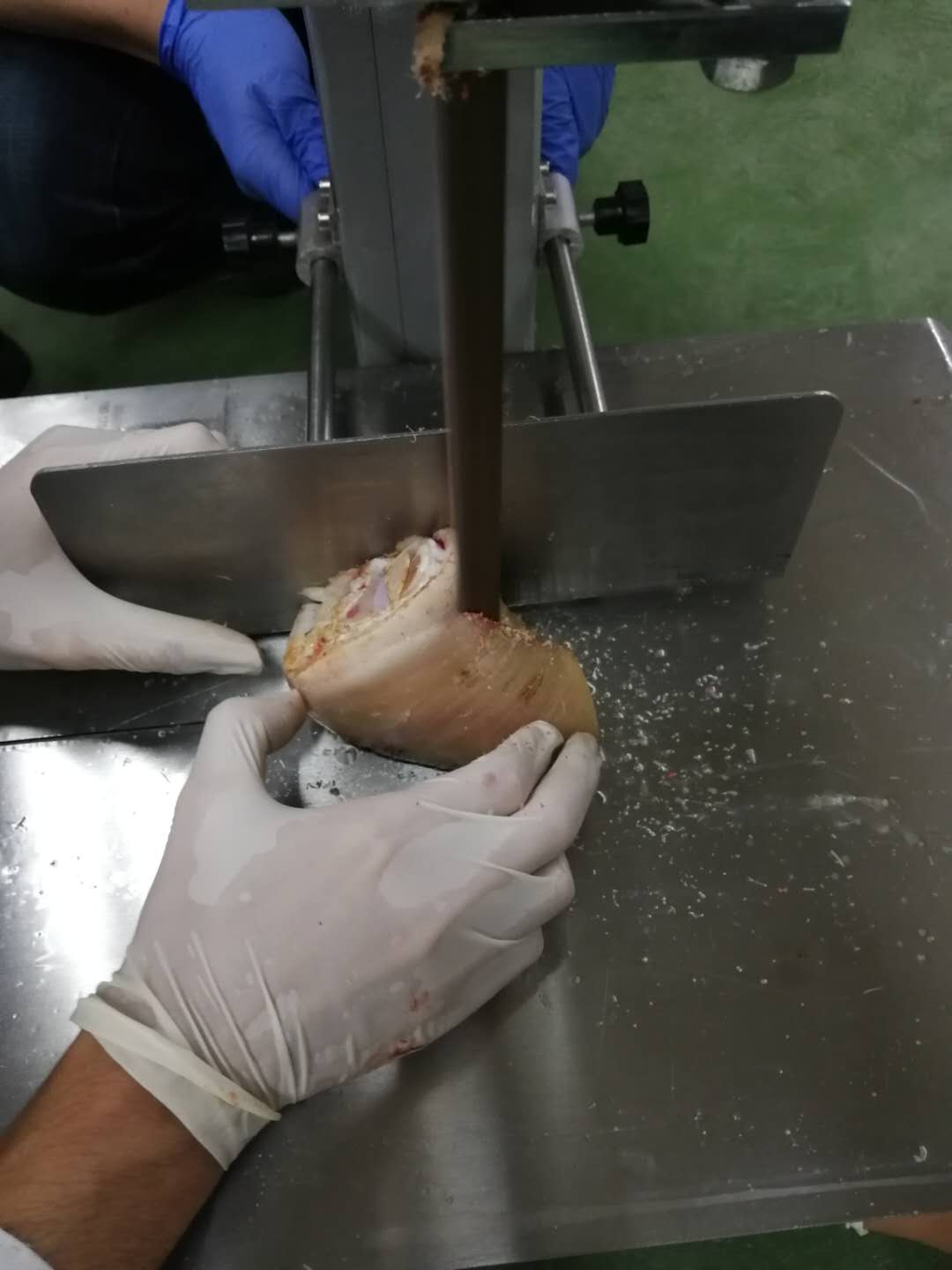

Supplement: Supplementary file 6 [file Image_3.JPEG]

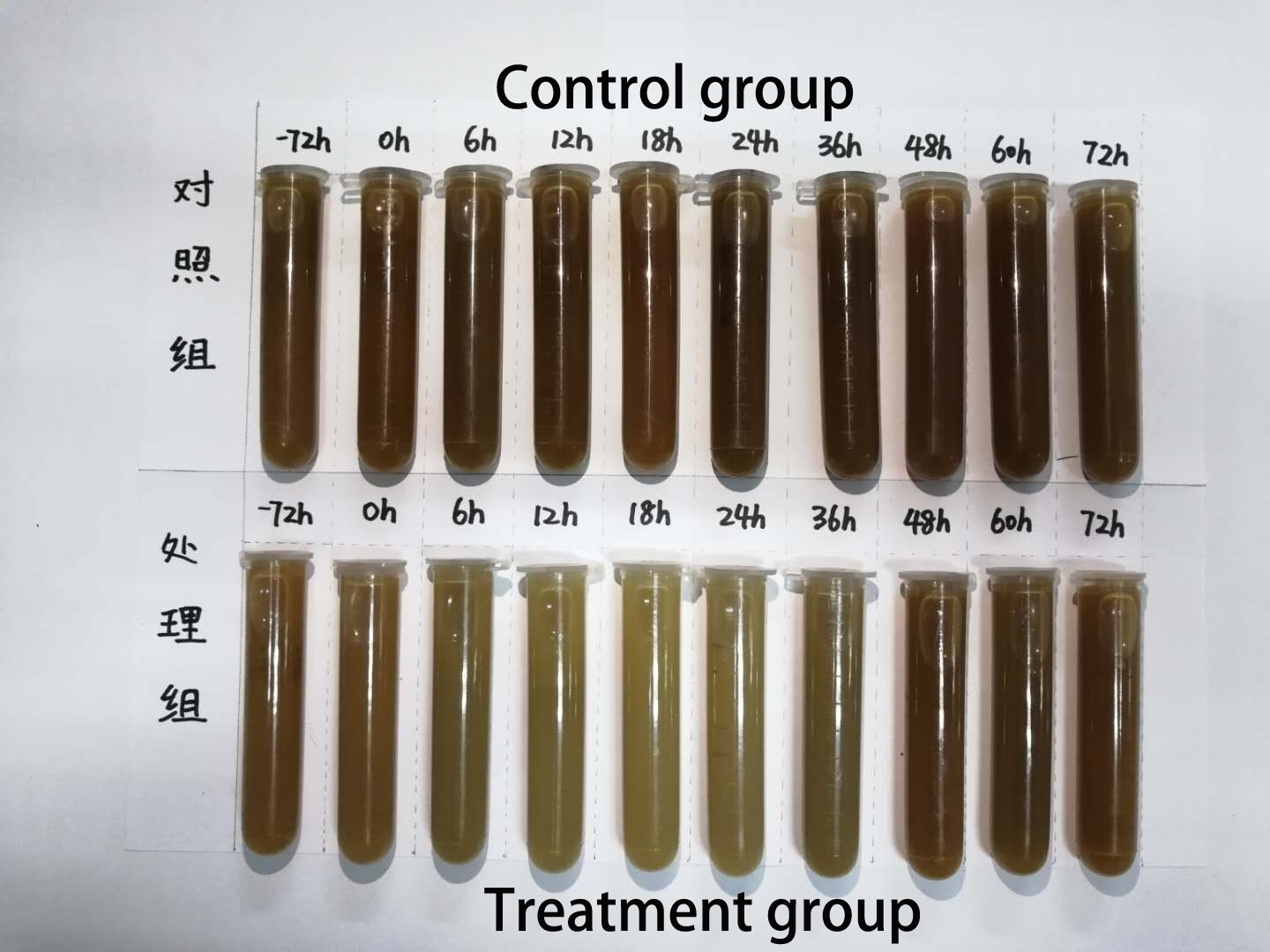

Supplement: Supplementary file 7 [file Image_4.JPEG]
